# Supplementary figures and images for: Kindlin2 enables EphB/ephrinB bi-directional signaling to support vascular development
Source: Life Sci Alliance. 2022 Dec 27;6(3):e202201800. doi: 10.26508/lsa.202201800 (PMC9795039; doi:10.26508/lsa.202201800)

**Fig. 2**

(B)

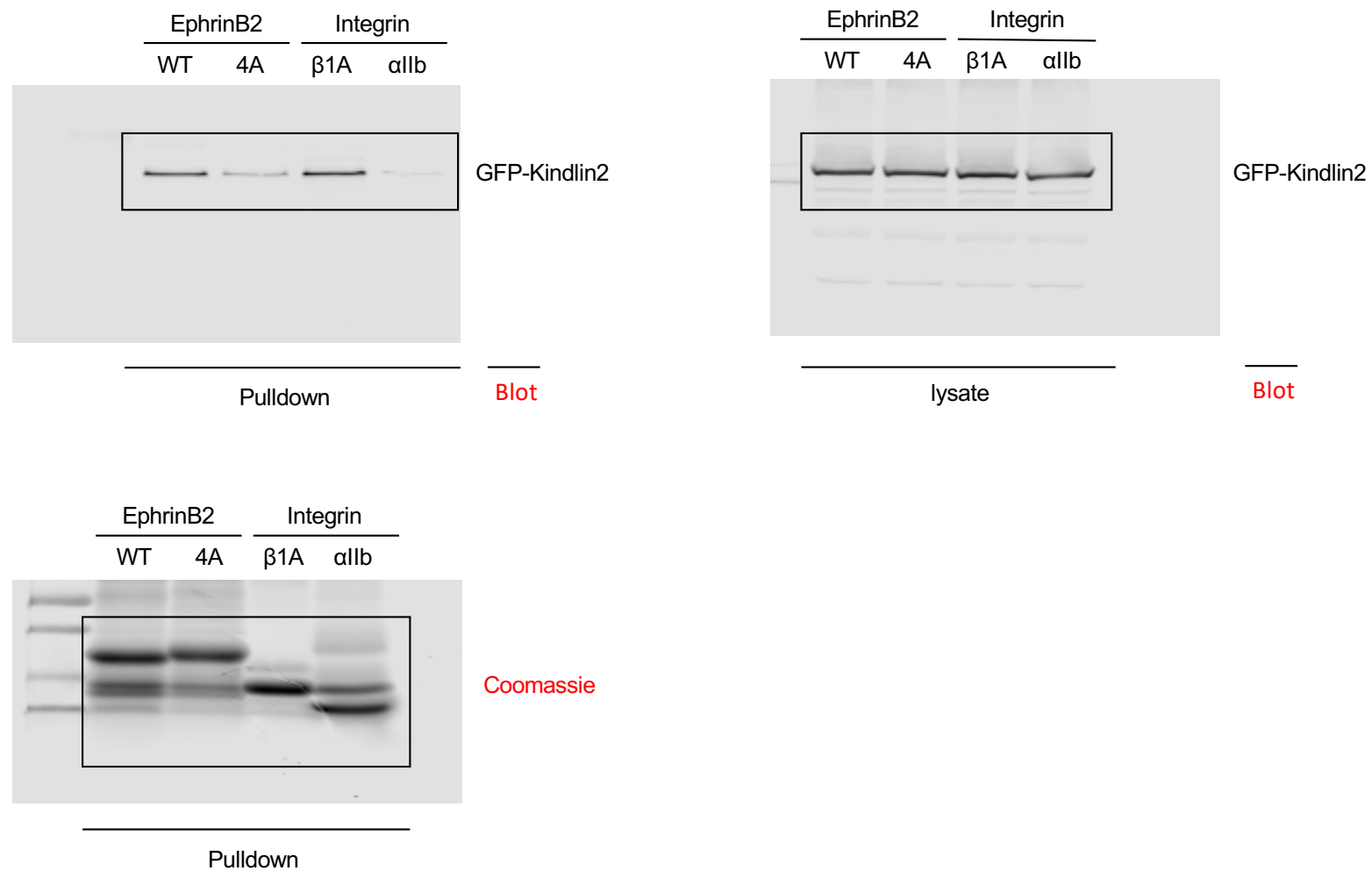

**Fig. 2**  
(C)

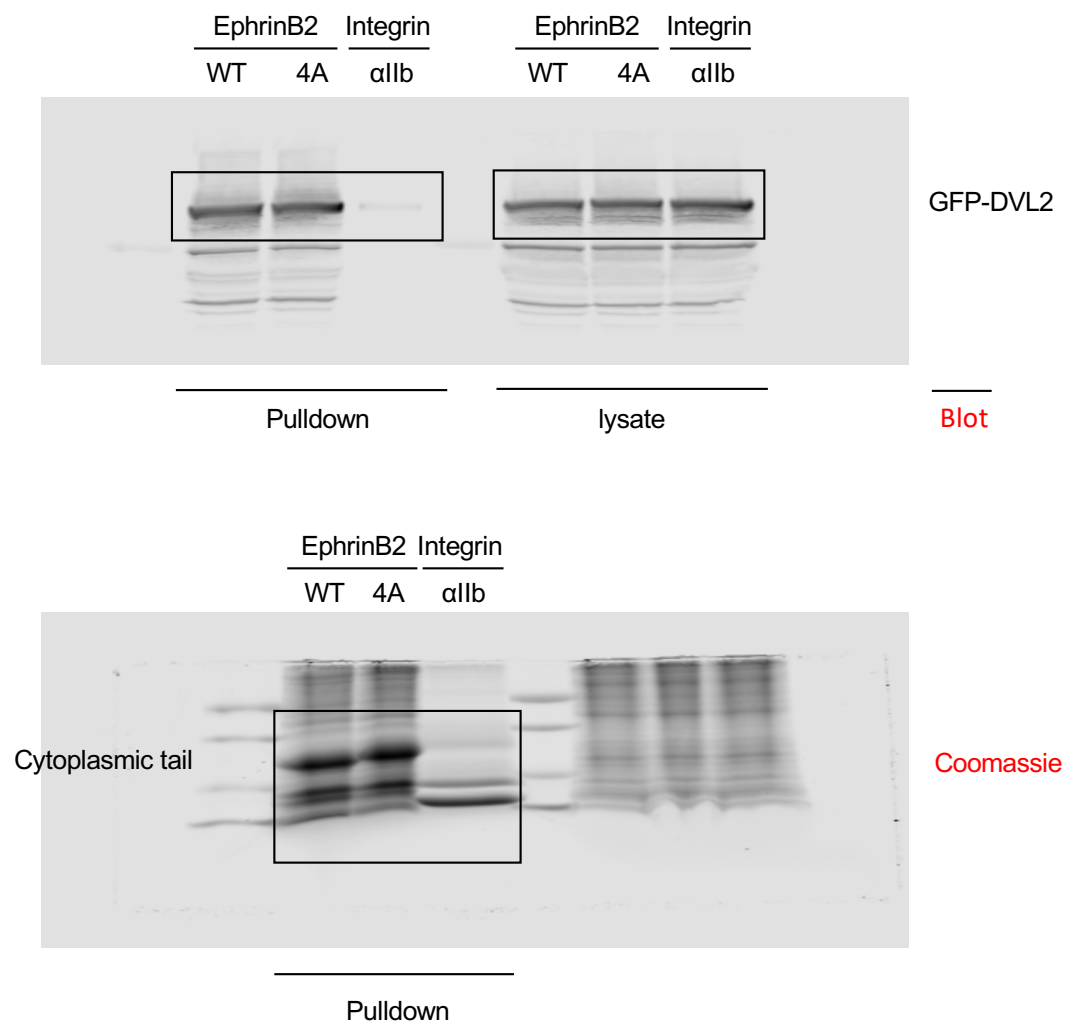

**Fig. 2**  
(D)

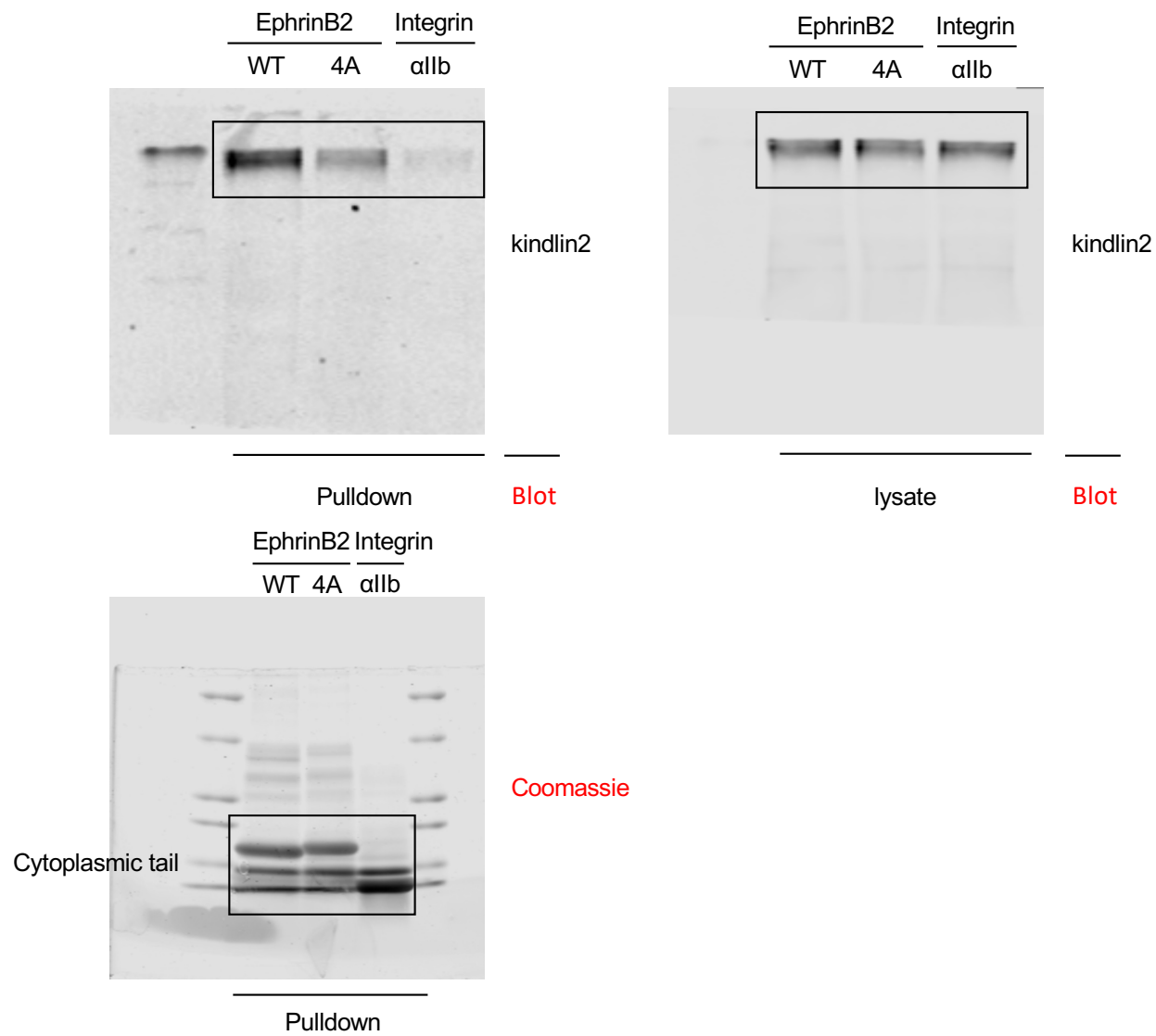

**Fig. 2**

(E)

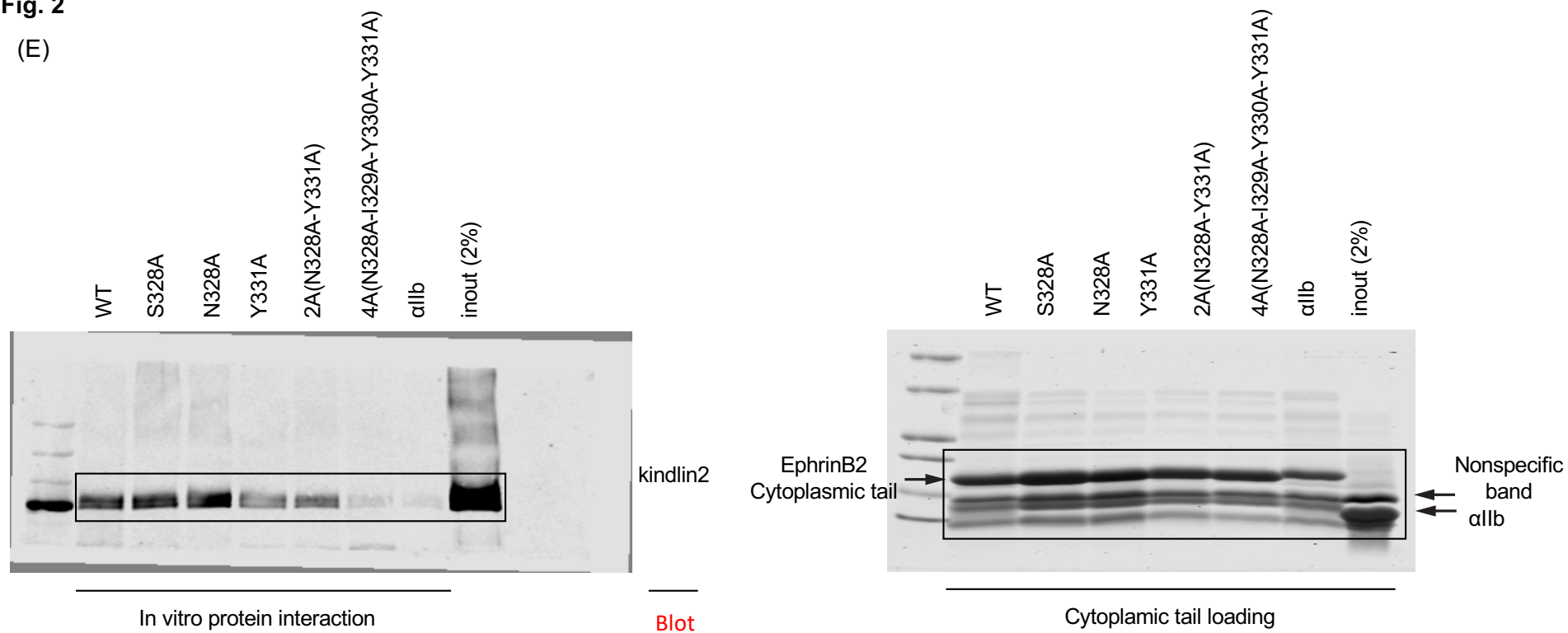

**Fig. 2**

(G)

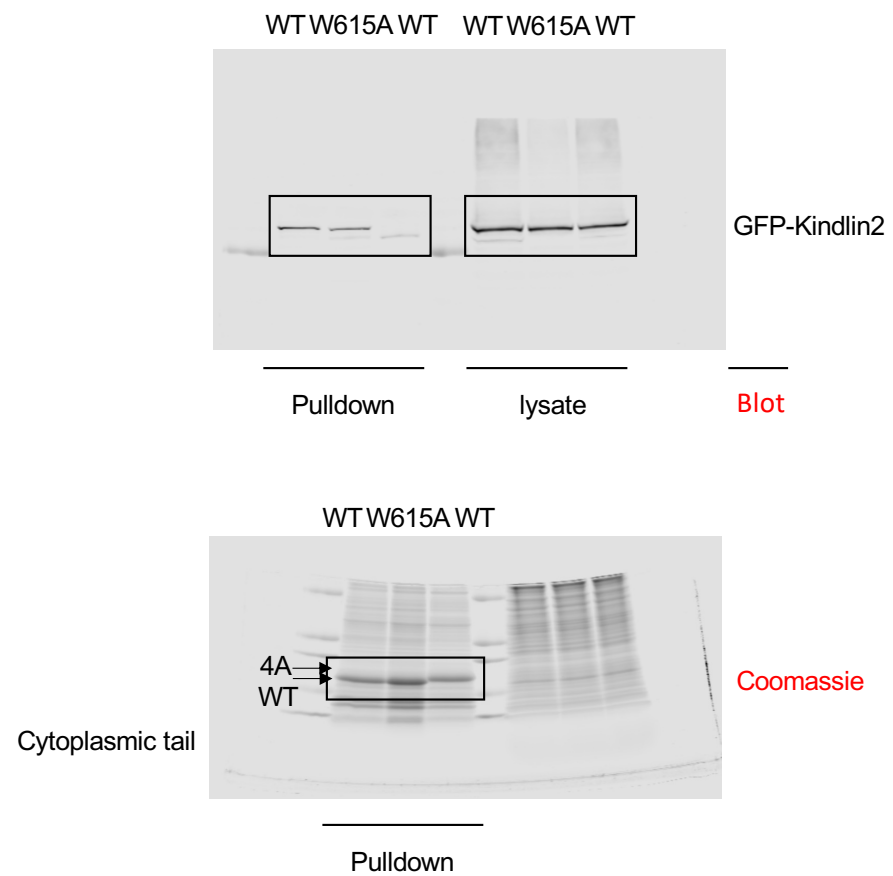

(H)

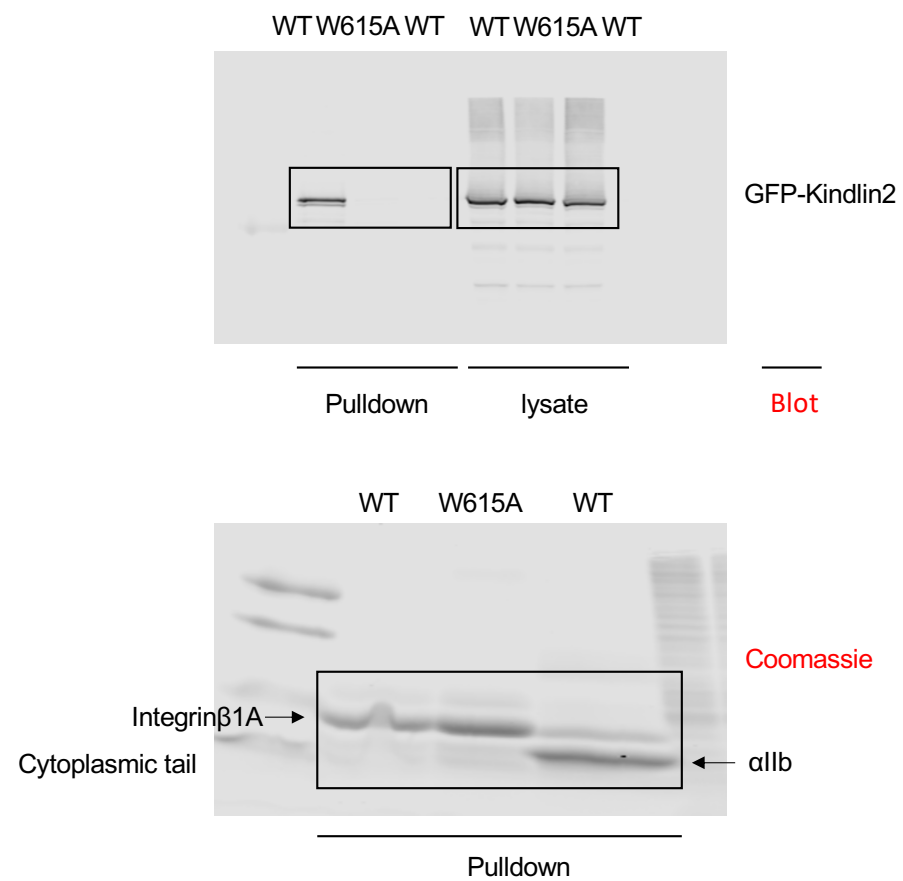

**Fig. 2**

(I)

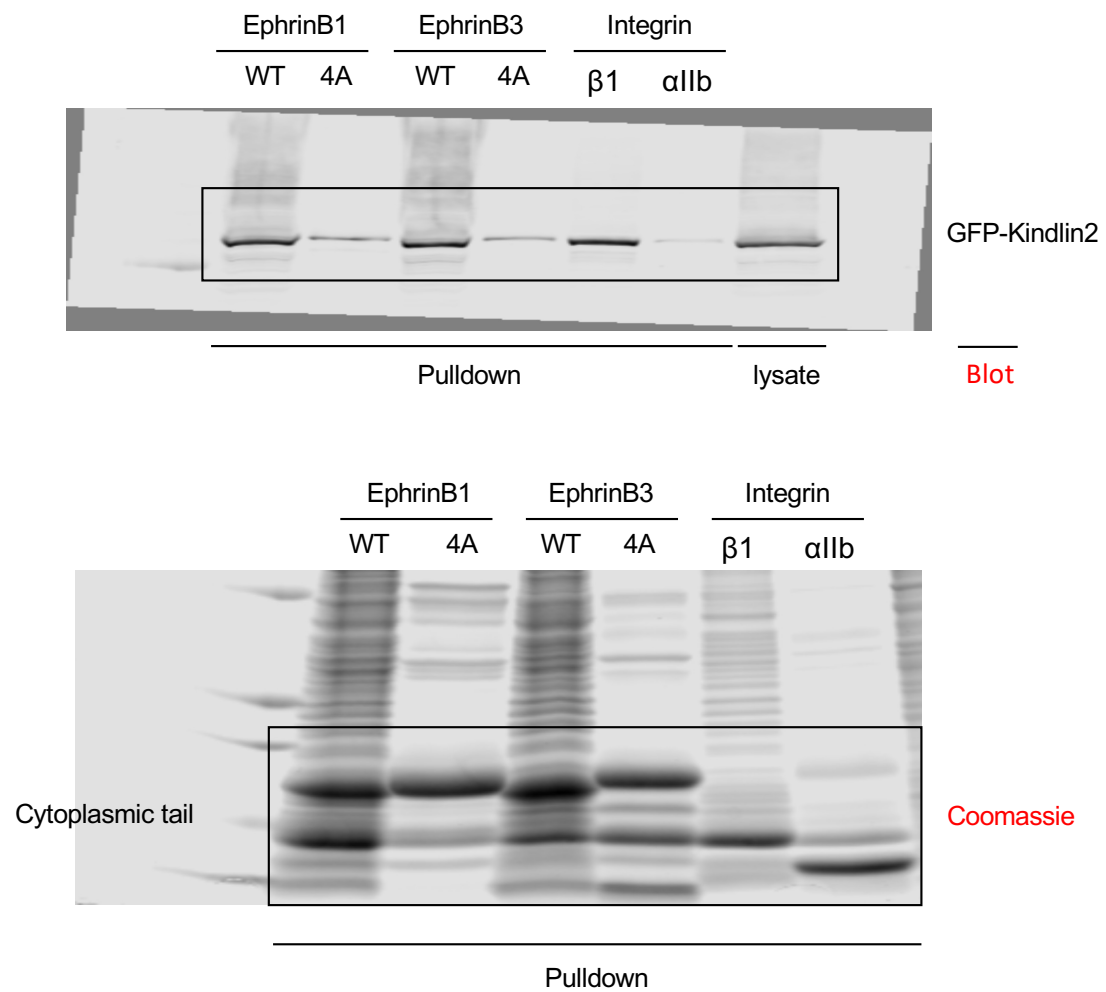

**Fig. 2**  
(K)

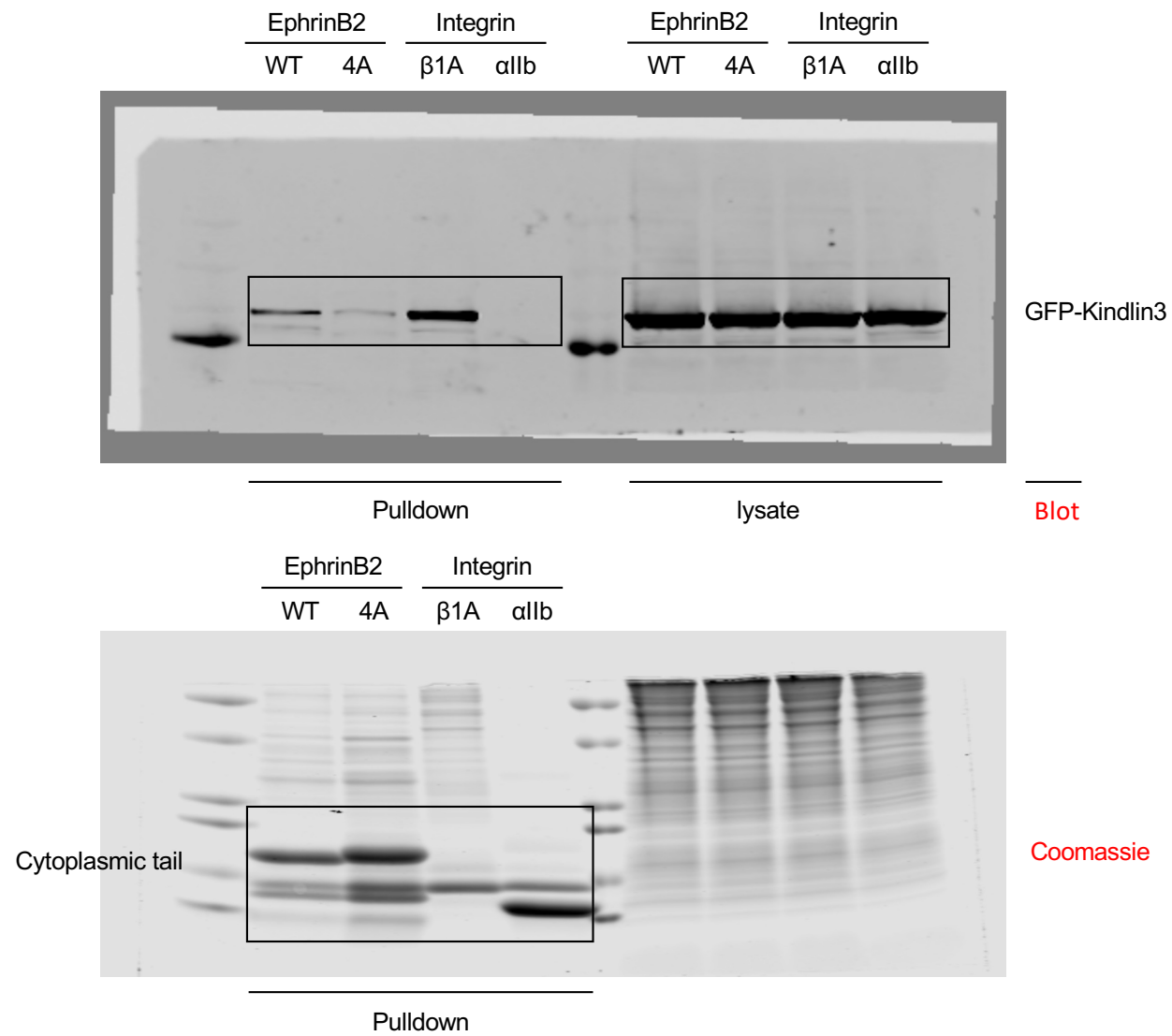

Supplement: Supplementary file 1 [file LSA-2022-01800_SdataF2.pdf]

**Fig. 3**

(C)

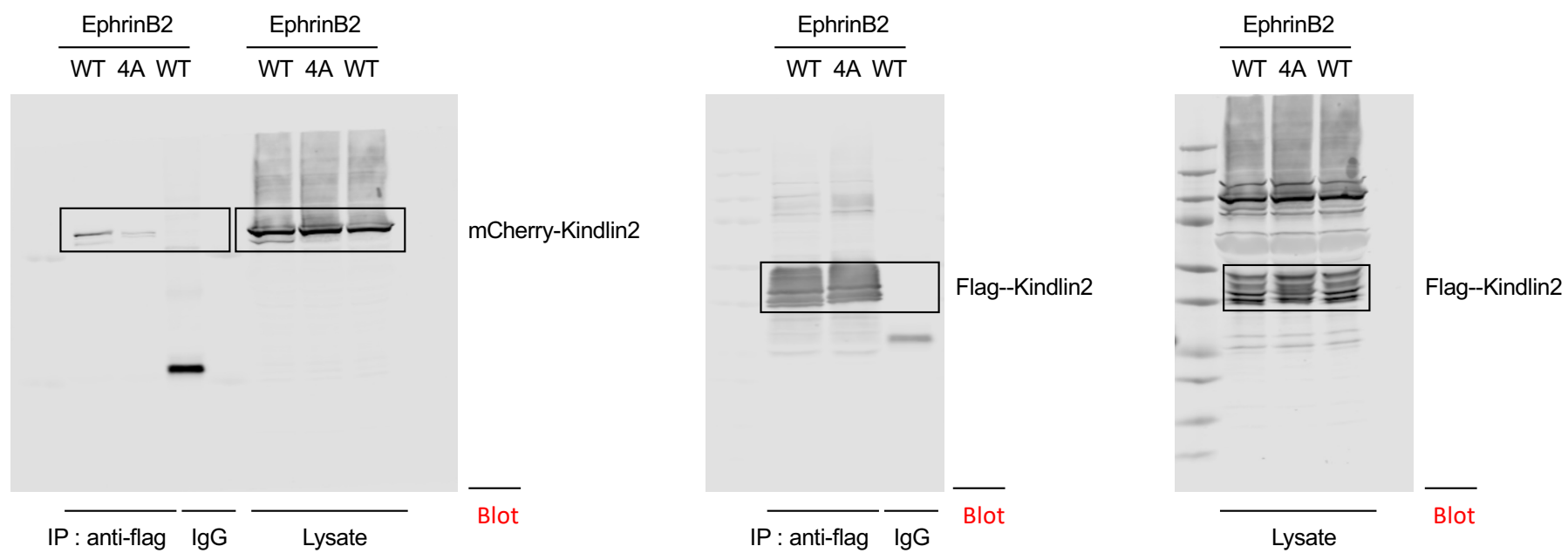

Supplement: Supplementary file 2 [file LSA-2022-01800_SdataF3.pdf]

**Fig. S5**

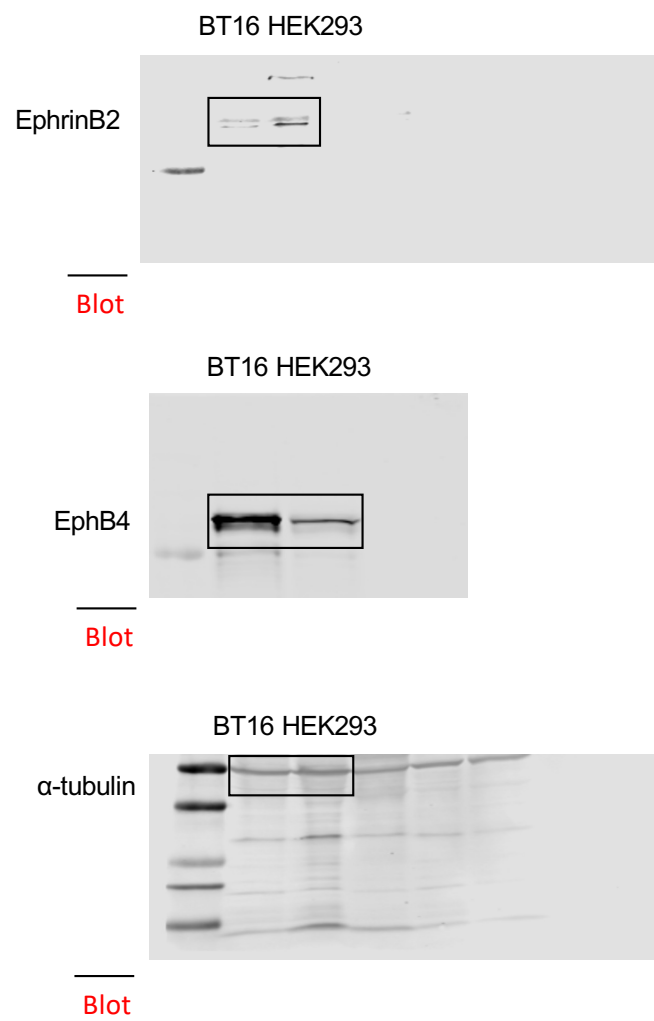

Supplement: Supplementary file 3 [file LSA-2022-01800_SdataFS4.pdf]

**Fig. 4**  
(B)

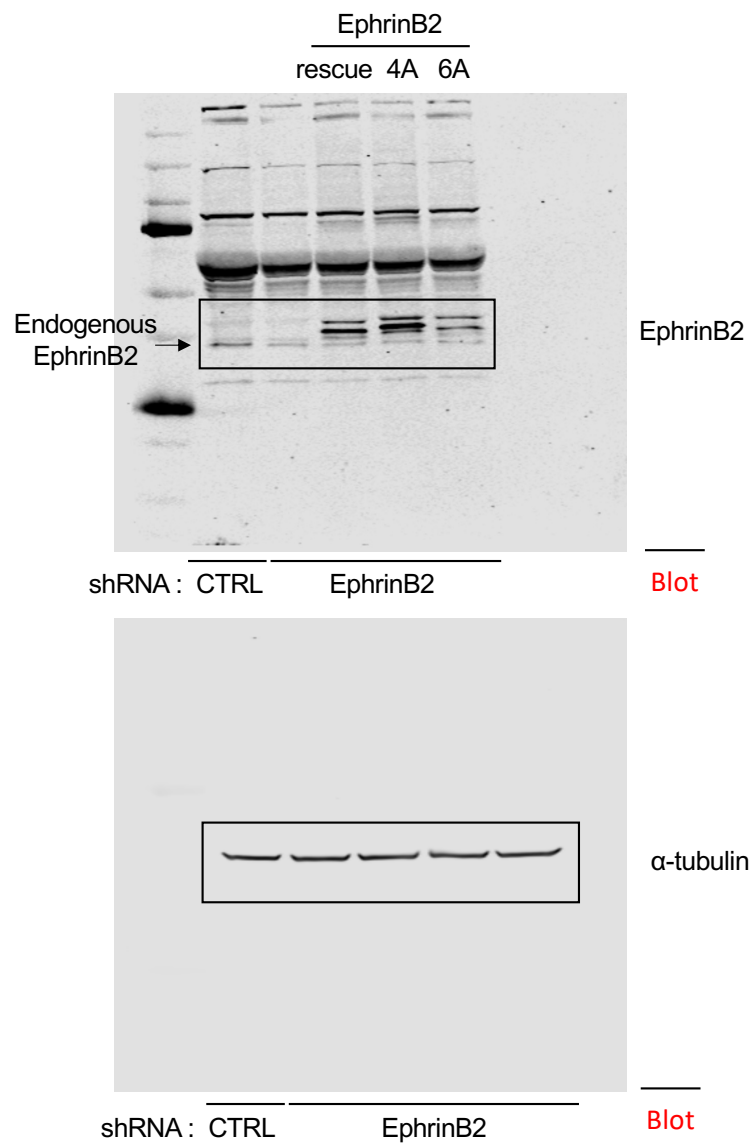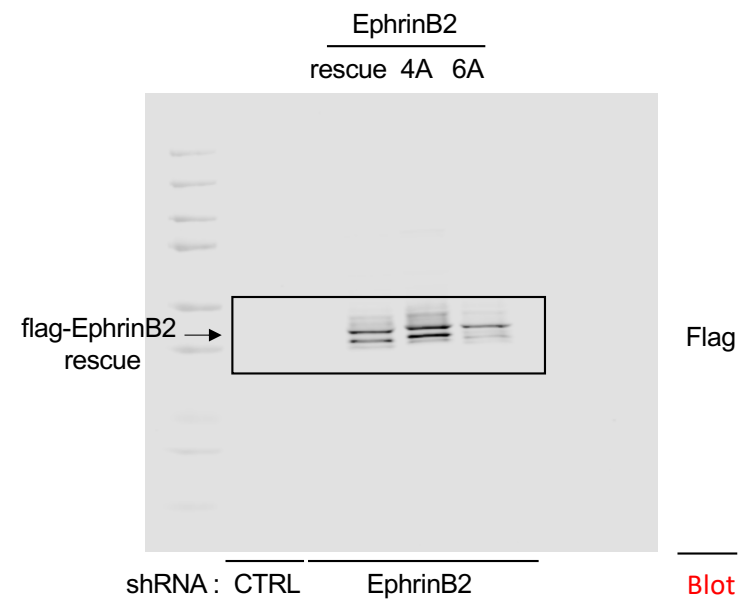

**Fig. 4**

(C)

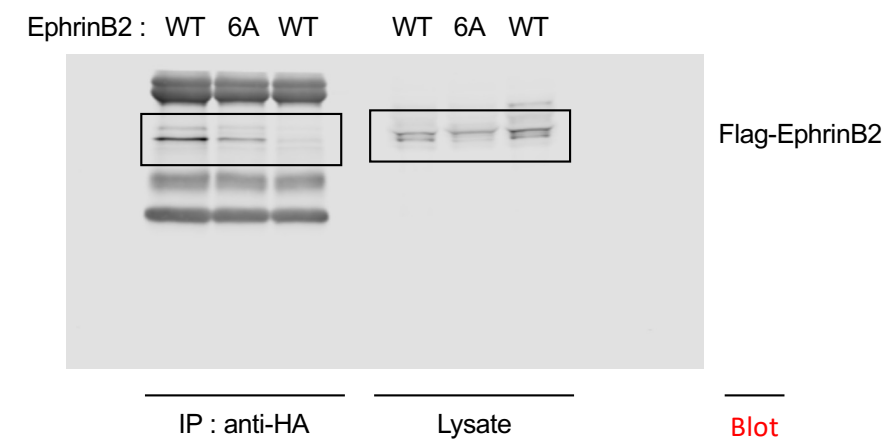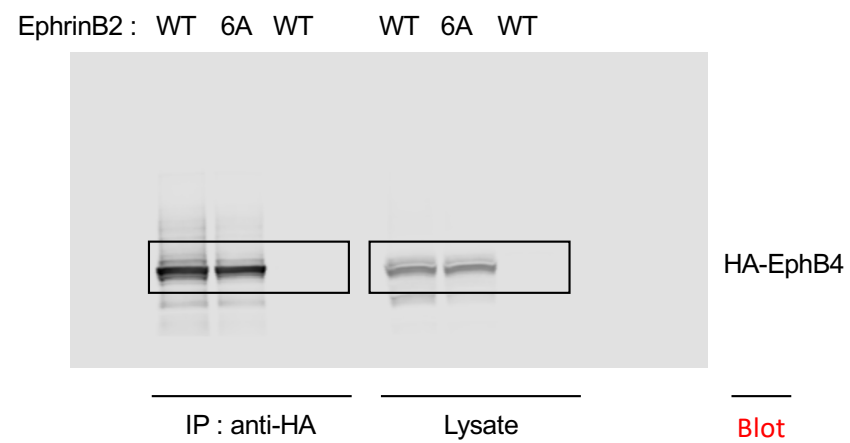

**Fig. 4**  
(E)

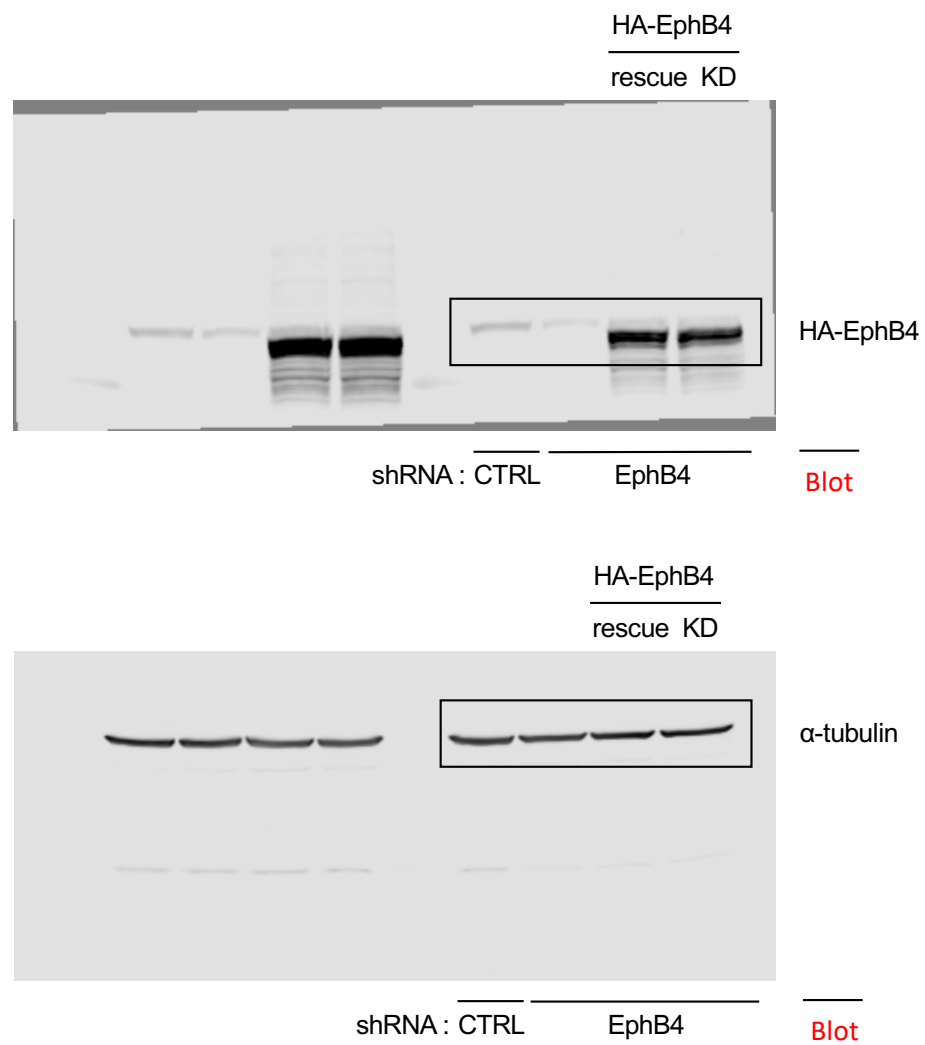

Supplement: Supplementary file 4 [file LSA-2022-01800_SdataF4.pdf]

**Fig. S4**

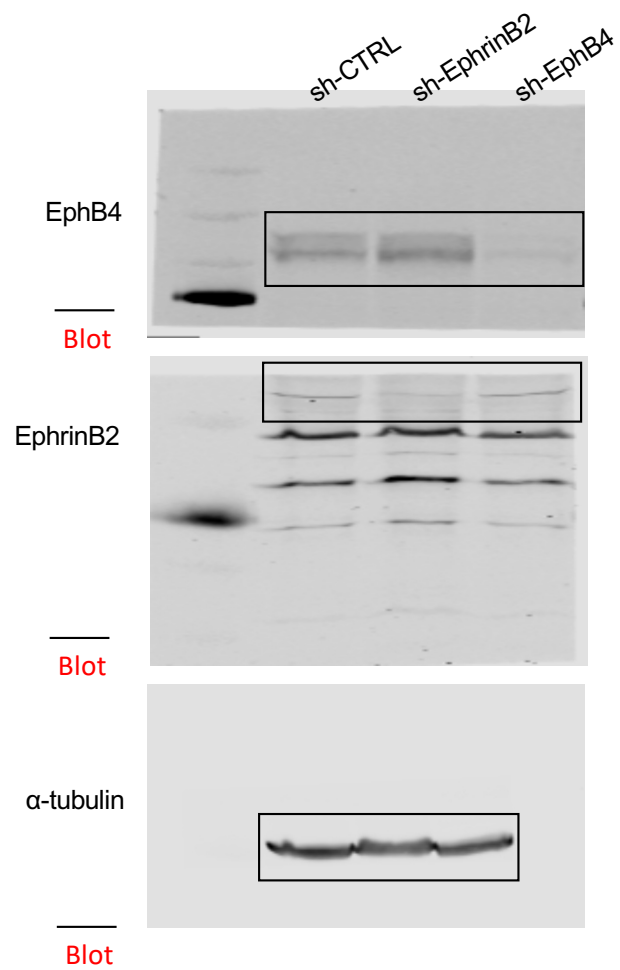

Supplement: Supplementary file 5 [file LSA-2022-01800_SdataFS5.pdf]

**Fig. 5**

(A)

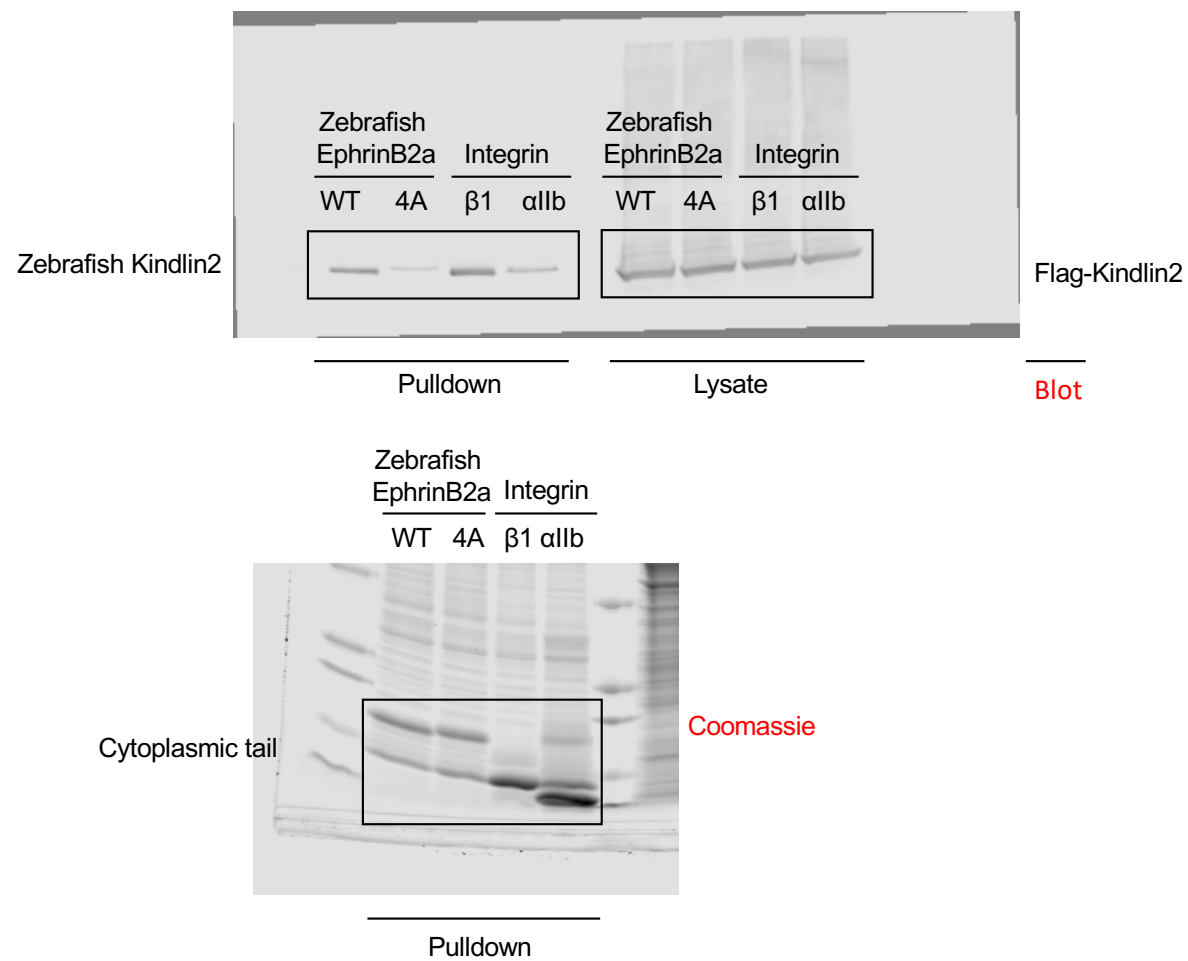

Supplement: Supplementary file 6 [file LSA-2022-01800_SdataF5.pdf]

**Fig. S6**

(A)

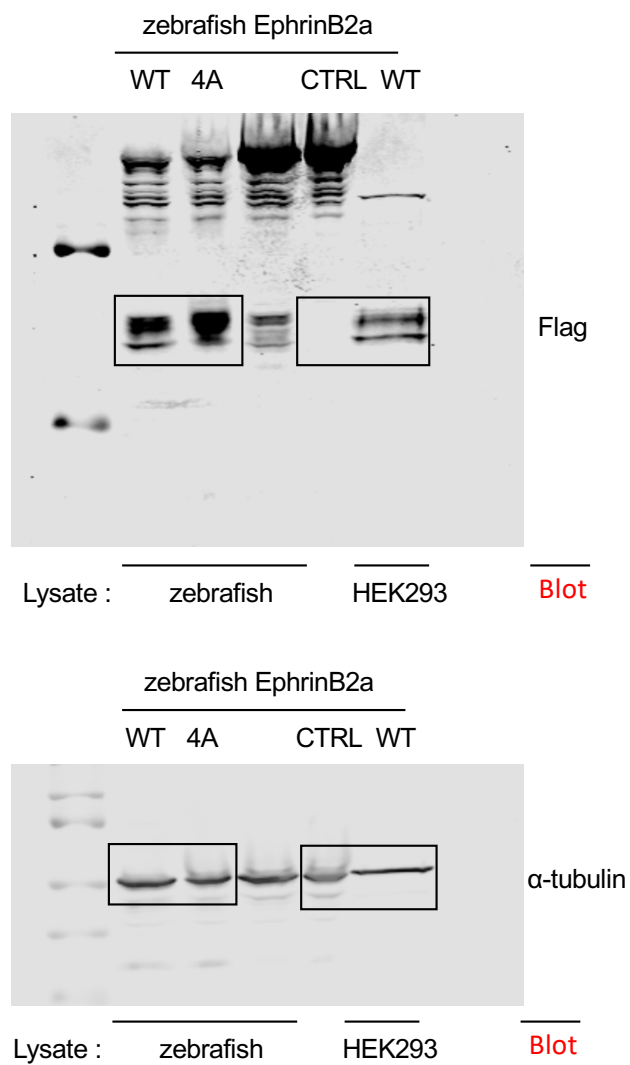

(B)

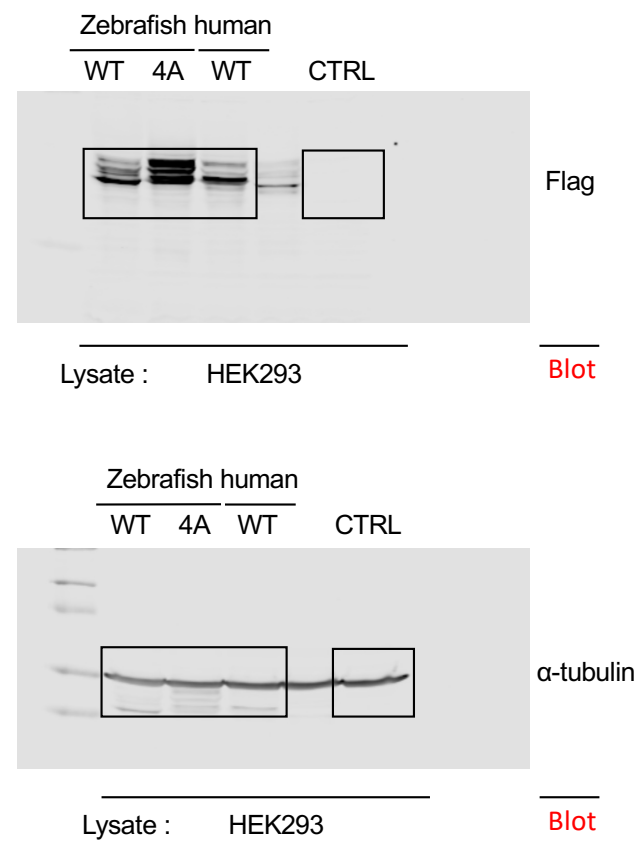

Supplement: Supplementary file 12 [file LSA-2022-01800_SdataFS6.pdf]

**Fig. 7**  
(E)

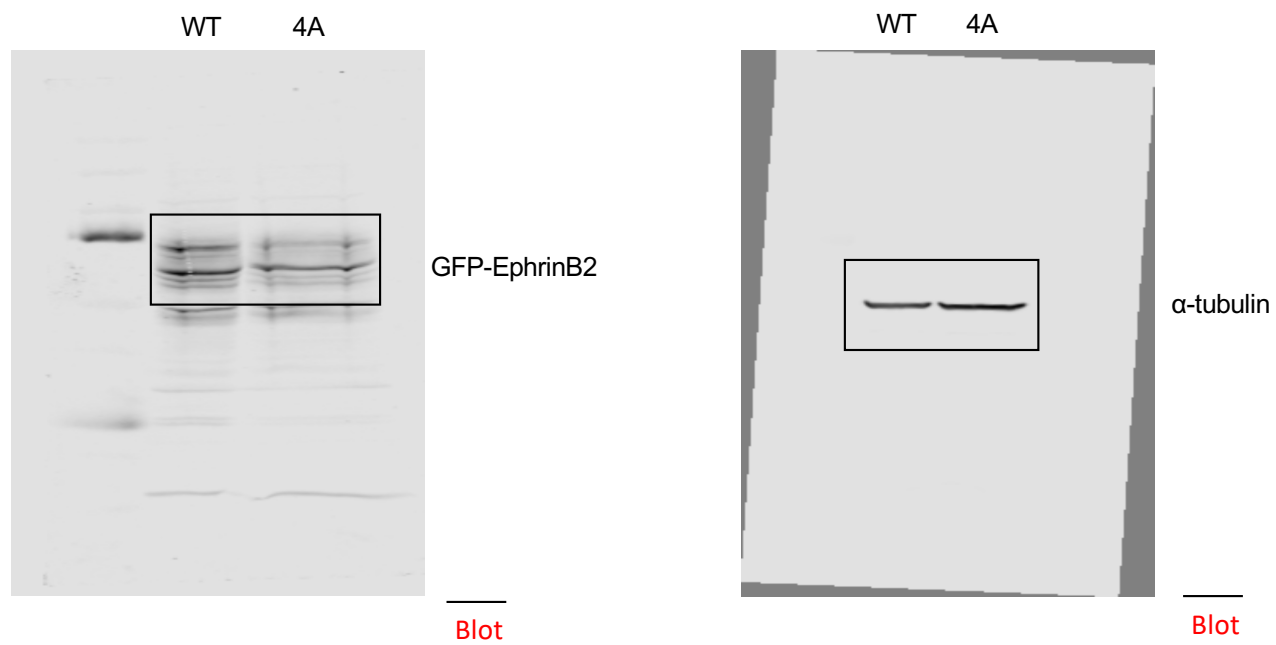

Supplement: Supplementary file 14 [file LSA-2022-01800_SdataF7.pdf]
