# Supplementary material for: Kindlin2 enables EphB/ephrinB bi-directional signaling to support vascular development
Source: Life Sci Alliance. 2022 Dec 27;6(3):e202201800. doi: 10.26508/lsa.202201800 (PMC9795039; doi:10.26508/lsa.202201800)
Supplement: Supplementary file 13 [file LSA-2022-01800_SdataF6.pdf]

Fig. 6

(B)

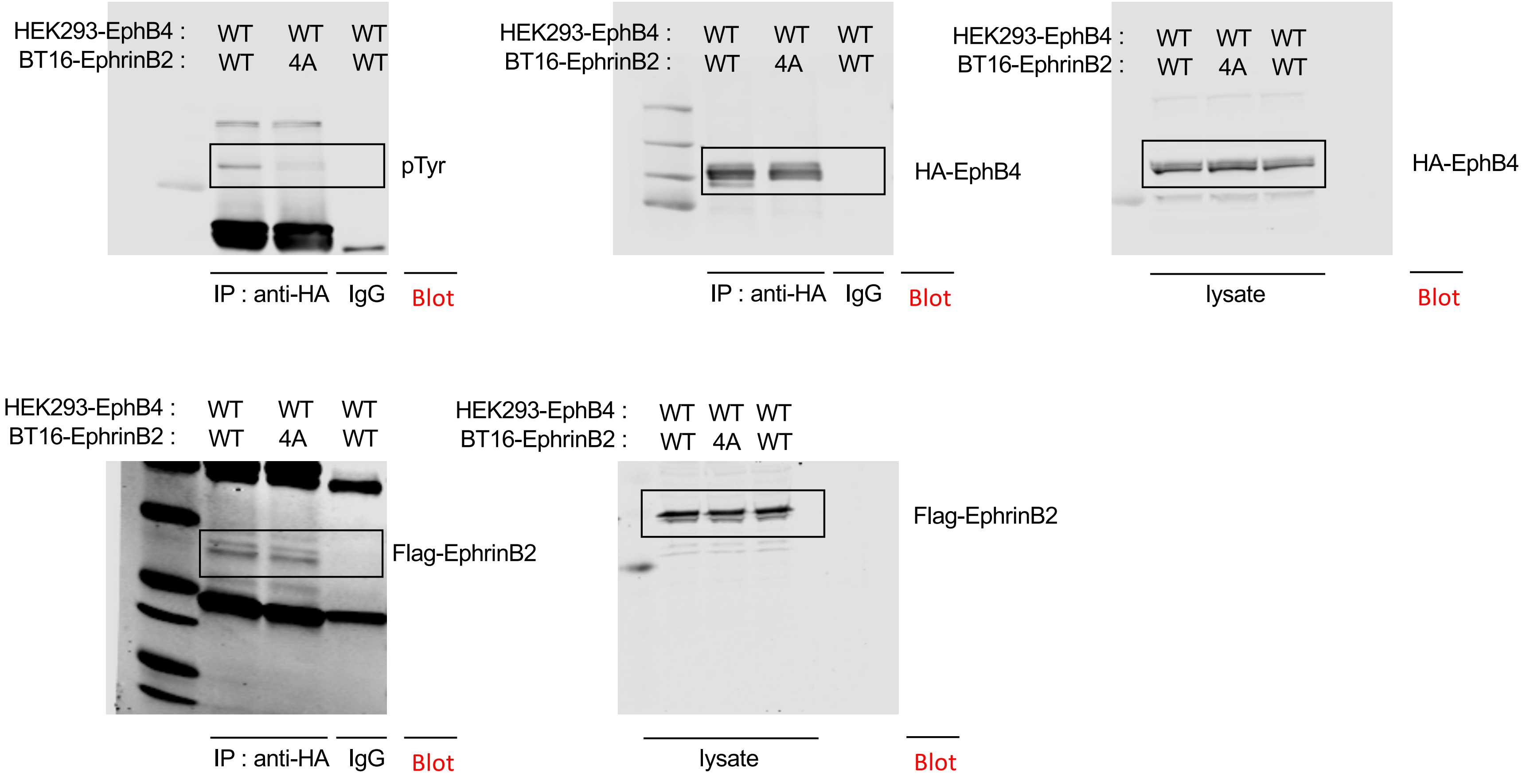

HEK293-EphB4 : WT WT WT
BT16-EphrinB2 : WT 4A WT
pTyr
IP : anti-HA
IgG
Blot
HEK293-EphB4 : WT WT WT
BT16-EphrinB2 : WT 4A WT
HA-EphB4
IP : anti-HA
IgG
Blot
HEK293-EphB4 : WT WT WT
BT16-EphrinB2 : WT 4A WT
HA-EphB4
lysate
Blot
HEK293-EphB4 : WT WT WT
BT16-EphrinB2 : WT 4A WT
Flag-EphrinB2
IP : anti-HA
IgG
Blot
HEK293-EphB4 : WT WT WT
BT16-EphrinB2 : WT 4A WT
Flag-EphrinB2
lysate
Blot

Fig. 6

(E)

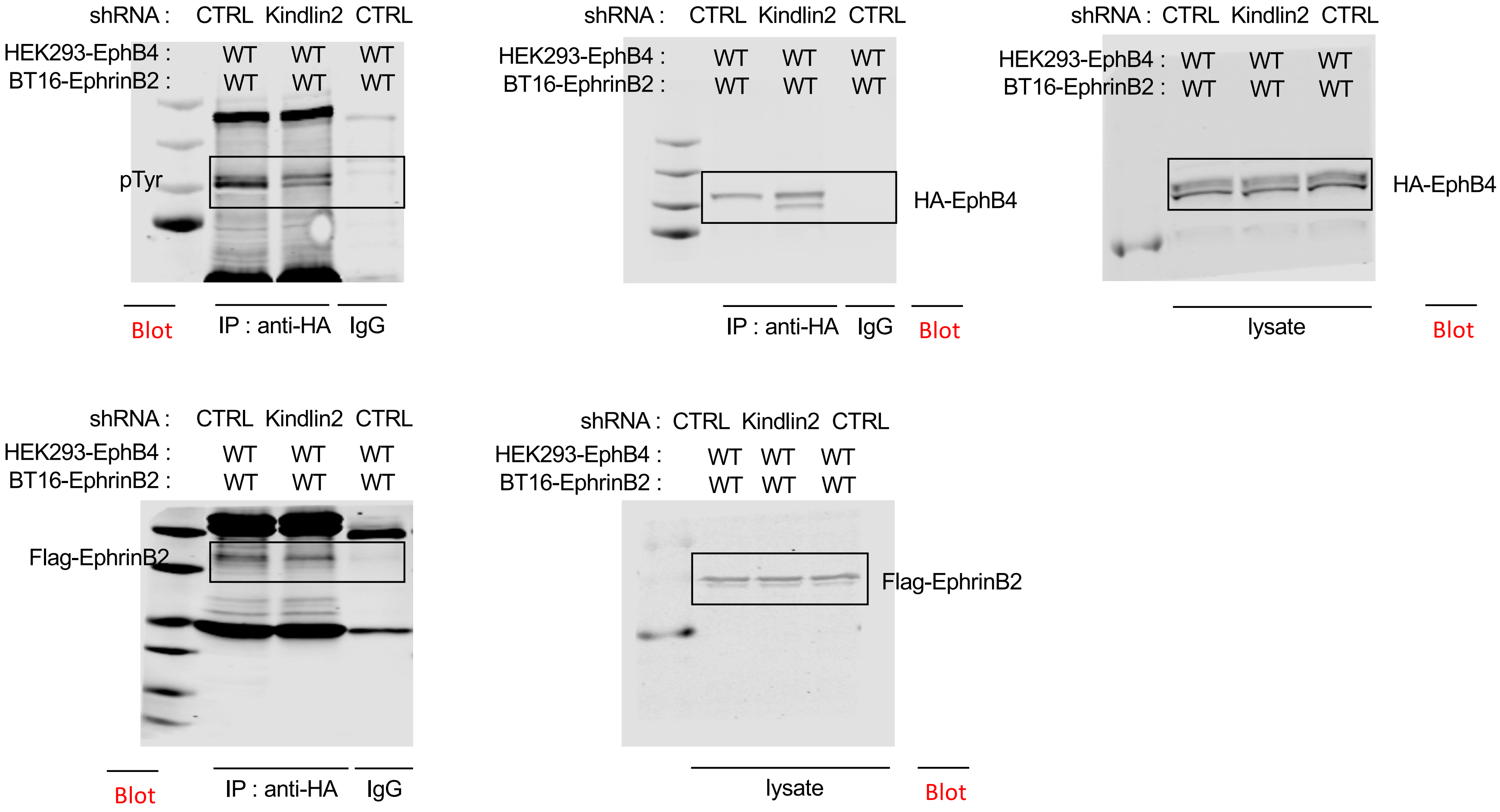
shRNA : CTRL Kindlin2 CTRL
HEK293-EphB4 : WT WT WT
BT16-EphrinB2 : WT WT WT
pTyr
Blot
IP : anti-HA
IgG
shRNA : CTRL Kindlin2 CTRL
HEK293-EphB4 : WT WT WT
BT16-EphrinB2 : WT WT WT
HA-EphB4
IP : anti-HA
IgG
Blot
shRNA : CTRL Kindlin2 CTRL
HEK293-EphB4 : WT WT WT
BT16-EphrinB2 : WT WT WT
HA-EphB4
lysate
Blot
shRNA : CTRL Kindlin2 CTRL
HEK293-EphB4 : WT WT WT
BT16-EphrinB2 : WT WT WT
Flag-EphrinB2
Blot
IP : anti-HA
IgG
shRNA : CTRL Kindlin2 CTRL
HEK293-EphB4 : WT WT WT
BT16-EphrinB2 : WT WT WT
Flag-EphrinB2
lysate
Blot

Fig. 6

(F)

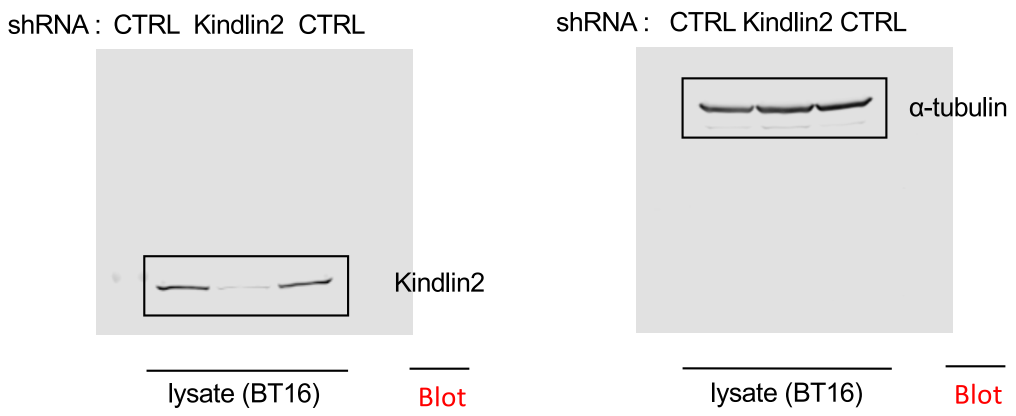
shRNA : CTRL Kindlin2 CTRL
Kindlin2
lysate (BT16)
Blot
shRNA : CTRL Kindlin2 CTRL
α-tubulin
lysate (BT16)
Blot

Fig. 6

(I)

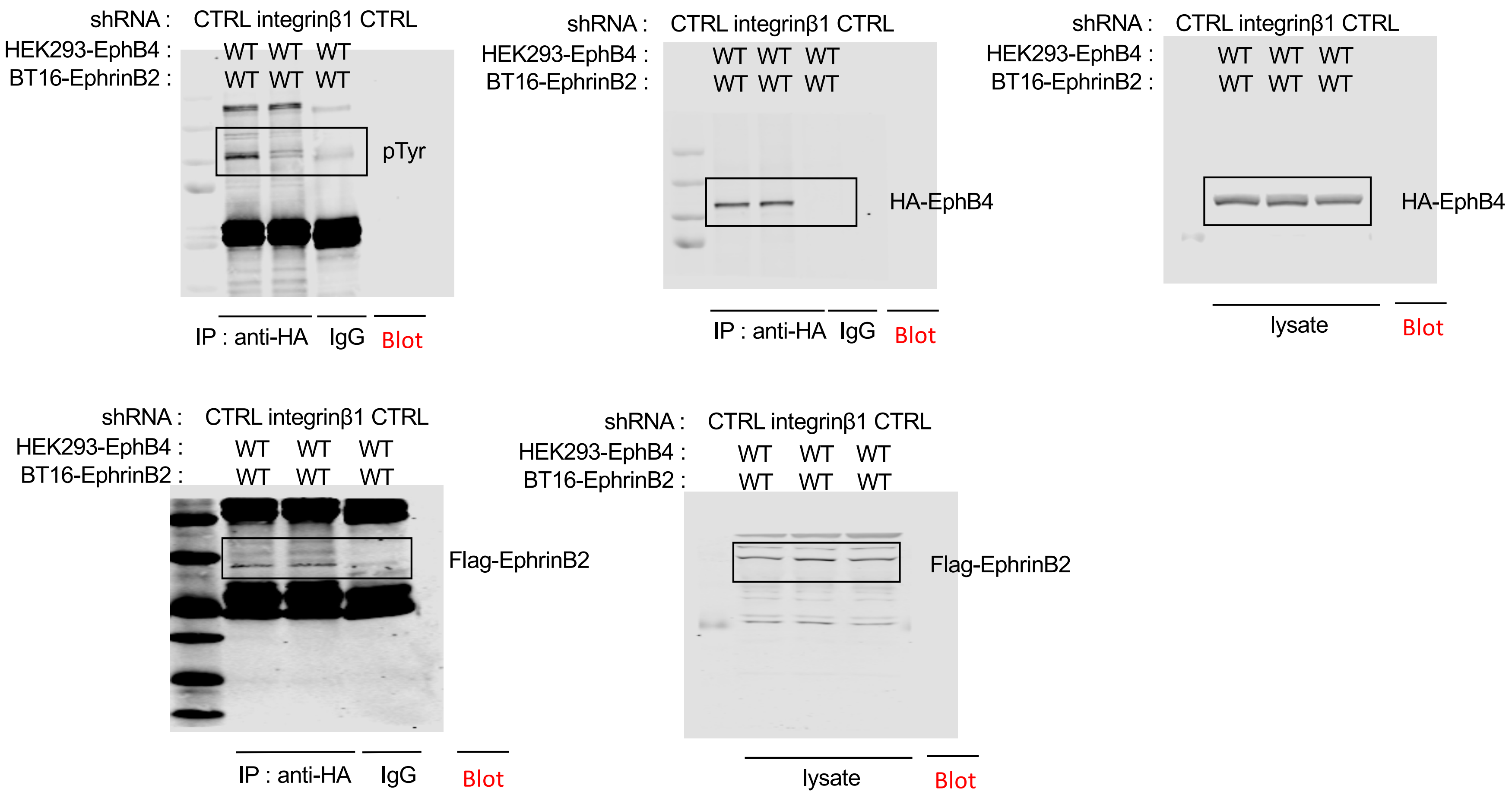
shRNA : CTRL integrinβ1 CTRL
HEK293-EphB4 : WT WT WT
BT16-EphrinB2 : WT WT WT
pTyr
IP : anti-HA IgG Blot
shRNA : CTRL integrinβ1 CTRL
HEK293-EphB4 : WT WT WT
BT16-EphrinB2 : WT WT WT
HA-EphB4
IP : anti-HA IgG Blot
shRNA : CTRL integrinβ1 CTRL
HEK293-EphB4 : WT WT WT
BT16-EphrinB2 : WT WT WT
HA-EphB4
lysate Blot
shRNA : CTRL integrinβ1 CTRL
HEK293-EphB4 : WT WT WT
BT16-EphrinB2 : WT WT WT
Flag-EphrinB2
IP : anti-HA IgG Blot
shRNA : CTRL integrinβ1 CTRL
HEK293-EphB4 : WT WT WT
BT16-EphrinB2 : WT WT WT
Flag-EphrinB2
lysate Blot

Fig. 6

(J)

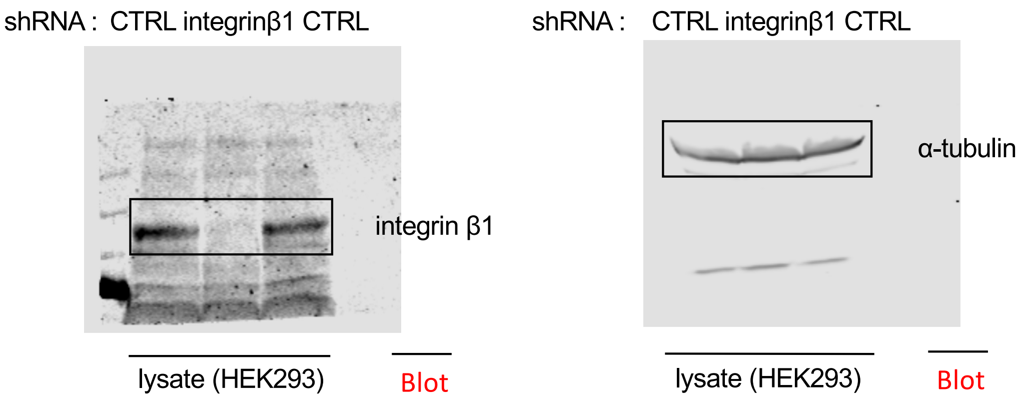

shRNA : CTRL integrinβ1 CTRL
integrin β1
lysate (HEK293)
Blot
shRNA : CTRL integrinβ1 CTRL
α-tubulin
lysate (HEK293)
Blot

Fig. 6

(M)

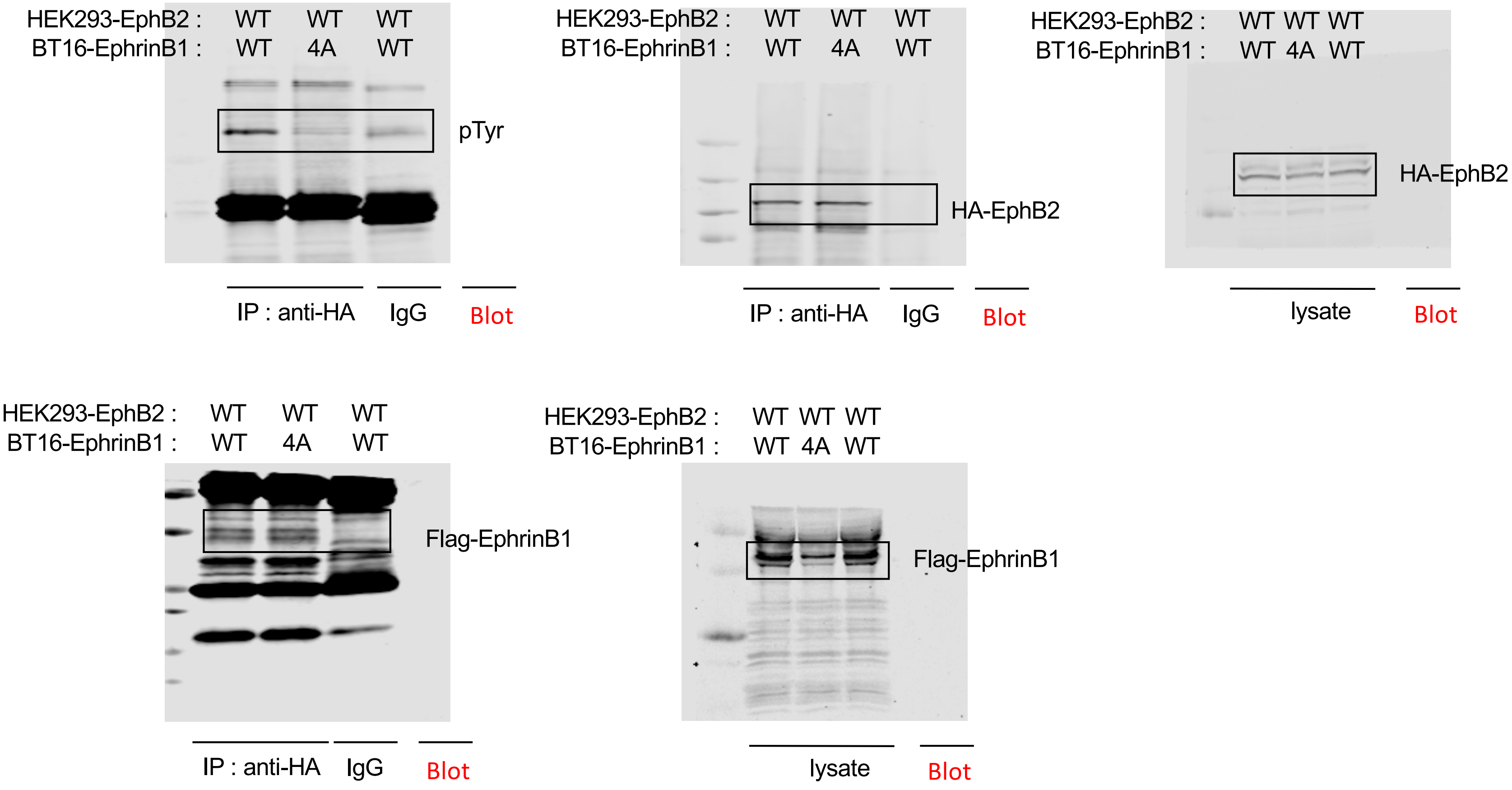
HEK293-EphB2 : WT WT WT
BT16-EphrinB1 : WT 4A WT
pTyr
IP : anti-HA
IgG
Blot
HEK293-EphB2 : WT WT WT
BT16-EphrinB1 : WT 4A WT
HA-EphB2
IP : anti-HA
IgG
Blot
HEK293-EphB2 : WT WT WT
BT16-EphrinB1 : WT 4A WT
HA-EphB2
lysate
Blot
HEK293-EphB2 : WT WT WT
BT16-EphrinB1 : WT 4A WT
Flag-EphrinB1
IP : anti-HA
IgG
Blot
HEK293-EphB2 : WT WT WT
BT16-EphrinB1 : WT 4A WT
Flag-EphrinB1
lysate
Blot
